# Construction of stable packaging cell lines for clinical lentiviral vector production

**DOI:** 10.1038/srep09021

**Published:** 2015-03-12

**Authors:** Khaled S. Sanber, Sean B. Knight, Sam L. Stephen, Ranbir Bailey, David Escors, Jeremy Minshull, Giorgia Santilli, Adrian J. Thrasher, Mary K. Collins, Yasuhiro Takeuchi

**Affiliations:** 1 Division of Infection and Immunity, University College London, London, UK; 2 Institute of Child Health, University College London, London, UK; 3 National Institute for Biological Standards and Control, South Mimms, UK; 4 DNA2.0, Menlo Park, CA94025, USA

## Abstract

Lentiviral vectors are useful experimental tools for stable gene delivery and have been used to treat human inherited genetic disorders and hematologic malignancies with promising results. Because some of the lentiviral vector components are cytotoxic, transient plasmid transfection has been used to produce the large batches needed for clinical trials. However, this method is costly, poorly reproducible and hard to scale up. Here we describe a general method for construction of stable packaging cell lines that continuously produce lentiviral vectors. This uses Cre recombinase-mediated cassette exchange to insert a codon-optimised HIV-1 Gag-Pol expression construct in a continuously expressed locus in 293FT cells. Subsequently Rev, envelope and vector genome expression cassettes are serially transfected. Vector titers in excess of 10^6^ transducing units/ml can be harvested from the final producer clones, which can be increased to 10^8^ TU/ml by concentration. This method will be of use to all basic and clinical investigators who wish to produce large batches of lentiviral vectors.

Lentiviral vectors (LV) have been used by many investigators to modify cells in vitro and in vivo, because they can integrate a transgene or shRNA into the genome of most cell types^1^. This work has extended to clinical trials using LV to modify bone marrow stem cells from patients with inherited genetic disorders; subsequent transplantation of the modified cells has resulted in clinical benefit for several severe conditions^2^,^3^. LV-modified autologous T cells have also been used in clinical trials to treat malignancies yielding encouraging results^4^.

As LV are replication defective, they need to be produced by co-expression of their constituents in one producer cell. These constituents are usually provided in three or four separate plasmids. The Gag-Pol expression cassette encodes HIV structural proteins and enzymes. Another cassette encodes Rev, which is an HIV accessory protein necessary for vector genome nuclear export. A third cassette encodes a heterologous envelope protein, often that of the G protein from vesicular stomatitis virus (VSV-G), that allows LV particle entry into target cells. Another cassette encodes the vector genome itself, which carries signals for incorporation into particles and an internal promoter driving transgene expression. The construction of stable packaging cell lines expressing all these components at high levels has been challenging. Notably, the HIV Gag-Pol cassette has proved impossible to express continuously at high level by plasmid transfection followed by antibiotic selection for plasmid integration. The cytotoxicity of Gag-Pol proteins has been suggested as a possible cause for this problem^5^,^6^. The commonly used VSV-G envelope is also cytotoxic^7^. Therefore, most LV batches, including those used in clinical trials to date, have been produced by transient transfection of HEK293T cells with multiple plasmids^8^,^9^,^10^,^11^,^12^,^13^,^14^. Such transfection is expensive, hard to reproduce at large scale, and results in contamination of the LV preparation with plasmids and cellular debris^15^.

LV production by stable producer cell lines (PCLs) would avoid some of these problems and will be particularly necessary to produce batches of LV for larger clinical trials and future gene medicines. As an alternative to continuous, constitutive vector production, inducible PCLs have been developed wherein inducible cassettes are used to express packaging functions. Only one of the reported inducible HIV-based PCLs, named GPRG, has been proposed for the production of therapeutic vectors for use in clinical trials targeting SCID-Xl^16^,^17^. Another inducible EIAV-based LV producer cell line has been developed to make therapeutic vectors for use in clinical trials targeting Parkinson's disease^18^,^19^. However, the scaling-up of inducible systems necessary for clinical-grade LV production is problematic, and additional purification steps of the vector preps to eliminate inducing agents are required. Furthermore, vector production rapidly declines as a result of instability of producer cell clones following induction^20^,^21^,^22^.

We previously constructed continuous, high-titer LV packaging cells called STAR^23^. To avoid the problem of VSV-G toxicity we used an envelope derived from the gammaretrovirus RD114, with the R-peptide cleavage site replaced with that of HIV-1 protease^23^. This mediates particularly efficient transduction of human hematopoietic stem cells (HSCs) and T cells^24^,^25^,^26^, which are important clinical gene therapy targets. We used gammaretroviral vectors (GRV) to express a codon-optimized HIV-1 Gag-Pol and Rev in STAR cells. This resulted in insertion of HIV-1 Gag-Pol in chromosomal loci that allowed its high-level, stable expression^23^. However, Gag-Pol and Rev expression could be lost in these cells as they were not maintained under antibiotic selection. There was also a possibility of packaging a GRV encoding Gag-Pol and Rev within LV particles making the vectors unsuitable for clinical application. Another constitutive PCL reported recently, RD2-MolPack^27^, used SIN LV to introduce HIV-1 tat and an RD114-derived envelope, which may pose safety concerns as these LVs might be packaged. It also has Gag-Pol and Rev in a single construct, which is another safety concern as it reduces the number of recombinations required to generate wild-type HIV. Furthermore, the method reported to introduce non-SIN LV into RD2-MolPack by transduction is not applicable to SIN LV, which are required for most gene therapy applications.

Here, we report a method that solves all these problems for generation of stable LV packaging and producer cell lines exemplified by the construction of WinPac cells. These cells are uniquely capable of producing self-inactivating (SIN) LVs at relatively high titers for prolonged periods.

## Results

### Constitutive HIV-1 Gag-Pol expression via recombinase mediated cassette exchange (RMCE)

**Figure 1:**
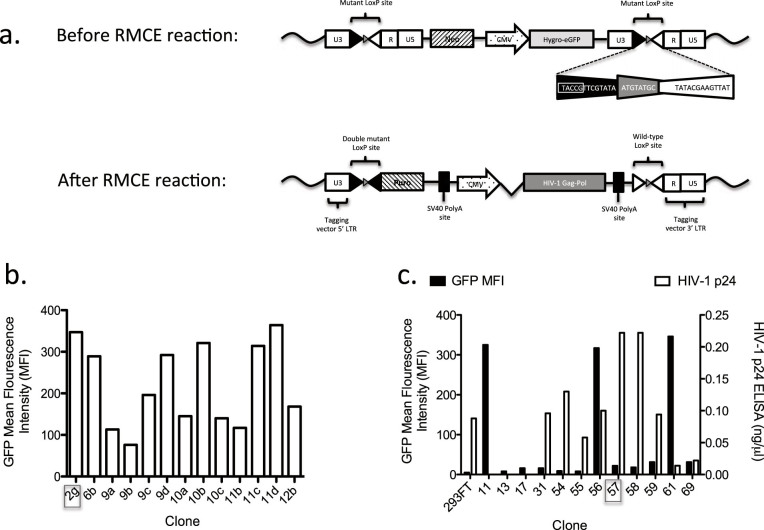
Stable expression of HIV-1 Gag-Pol in 293FT cells via Cre recombinase-mediated cassette exchange (RMCE). (a) Schematic representation of RMCE to introduce codon-optimized HIV-1 Gag-Pol at a transcriptionally active chromosomal locus. Neo, Neomycin resistance gene; CMV, Cytomegalovirus promoter; Hygro-eGFP, hygromycin-resistance gene and eGFP fusion transgene; Puro, puromycin resistance gene. (b) GFP mean fluorescence intensity (MFI) as determined by FACS analysis of hygromycin-resistant clones following transduction with the lox-P tagging MLV vector. The median MFI was 195 (range 78–359). Clone 2G (boxed) was chosen for further experiments based on the relatively high MFI, superior stability of MFI over 50 passages, and a single copy of the tagging vector by Q-PCR (data not shown). (c) Levels of HIV-1 p24 in culture supernatant of puromycin-resistant clones following the co-transfection of the Cre recombinase-encoding plasmind (pCAGGS Cre) and pSLS94 containing the HIV-1 Gag-Pol exchange cassette. The median p24 was 0.095 ng/μl (range 0–0.23). GFP MFI as determined by FACS analysis of puromycin-resistant clones is also shown. Clone 57 was chosen for further experiments based on highest p24 levels and loss of eGFP expression following RMCE reaction.

Traceability of clinical vector producer cell lines is likely to be required in the current good manufacturing practice (cGMP) regulation. Traceable 293FT cells with well-documented culture history^28^ were therefore used for packaging cell development. We designed a strategy to introduce a codon-optimized HIV-1 Gag-Pol by recombinase mediated cassette exchange^29^,^30^. This strategy takes advantage of GRV‘s ability to integrate within chromosomal loci that can support constitutive, high-level expression of HIV-1 Gag-Pol but minimizes GRV derived sequences remaining in the final packaging cells (Figure 1a). An MLV-based GRV encoding a Hygro-eGFP hybrid with an LE mutant LoxP site cloned into its 3′ U3 region, pSLS51 (Supplementary Figure S4a), was used to transduce 293FT cells at low MOI and the cells were then selected in hygromycin (Figure 1a Before RMCE). Among the hygromycin resistant clones we selected clone 2 g, which had a single vector copy per cell as determined by qPCR and stably expressed eGFP at a relatively high MFI for more than 50 passages (Figure 1b). The vector integration site was identified by inverse PCR as nucleotide position 10619185 in the first intron of midline 1 gene (MID1) on the X chromosome (GRCh38 assembly, 2013 Dec release), the vector was integrated in reverse orientation to the MID1 gene.

Subsequently, a plasmid encoding a codon-optimized H87Q Gag-Pol mutant driven by CMV promoter and flanked by RE mutant LoxP sites was constructed, pSLS94 (Supplementary Figure S4a). The H87Q mutant is a naturally occurring capsid mutation that enhances transduction of mouse and monkey cells without compromising transduction of human cells^31^,^32^,^33^. This can facilitate pre-clinical testing using these vectors. Additionally, there was a more extensive optimization in this current construct according to codon optimization indices, compared to our previous HIV-1 Gag-Pol construct used in STAR cells (Supplementary Table S1). A promoter-less puromycin resistance gene with a polyA signal was also cloned downstream from the 5′ RE mutant LoxP site. This plasmid (pSLS94) was co-transfected with a Cre-recombinase-encoding plasmid resulting in recombination between the integrated LE mutant LoxP sites and the RE mutant LoxP sites in pSLS94 (Figure 1a After RMCE). As a result, the Gag-Pol expression cassette was integrated between a double (LE + RE) mutant LoxP site and a wild type LoxP site. Since Cre-recombinase has low affinity to the former, the cassette will remain stably integrated^29^. Puromycin-resistant clones were then tested for successful Cre-mediated recombination by the absence of GFP expression (by flow cytometry) and HIV-1 Gag-Pol expression levels (by HIV-1 p24 ELISA). Clone 57 was chosen for further experiments as it had high level Gag-Pol expression and had lost GFP expression (Figure 1c).

### Establishment of a WinPac-RD packaging cell line: introduction of HIV-1 Rev and an RDpro envelope

**Figure 2:**
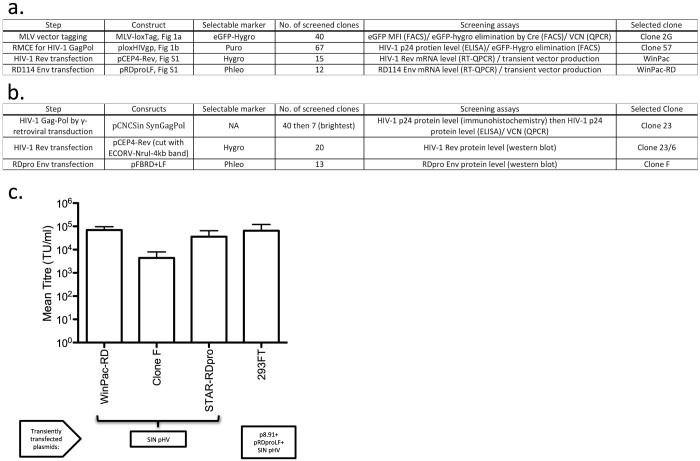
Establishment of (pre-)packaging cell line with a RD114-derived envelope. (a) Construction steps of WinPac-RD (pre-)packaging cell line. (b) Construction steps of an alternative (pre-)packaging cell line using self-inactivating MLV vector for Gag-Pol expression, Clone F (c) Trials of transient vector production. Transient transfection of SIN GFP-encoding HIV-1 vector plasmid in (pre-) packaging cell lines were compared with 3 plasmid transfection of parental 293FT cells. Data shown represents mean +/− SD from 8 independent experiments.

The remaining vector components were introduced by a series of plasmid DNA transfection, antibiotic selection, cell cloning and clone screening (Figure 2a). Firstly, HIV-1 Rev, which is required for nuclear export of vector genomes containing Rev response element (RRE), and secondly, RDpro envelope, derived form the gammaretroviral RD114 envelope glycoprotein^23^, were introduced. Cell clones at each step were screened for both RNA expression and vector production by transient transfection of missing vector components. The best performing clones, WinPac and WinPac-RD respectively, were selected for further study (described in detail in the supplementary information, Cell Construction and Figures S5 and S6). For comparison we included STAR cells with RDpro envelope (STAR-RDpro) as well as an alternative cell construct, Clone F (Figure 2b), in transient vector production assays (Figure 2c). Clone F was based on 293FT cells, like WinPac cells, and contains an HIV-1 Gag-Pol expression cassette introduced by a self-inactivating GRV proviruses to minimize the risk of Gag-Pol RNA packaging. WinPac-RD cells achieved a similar titer as the standard three plasmid transfection of 293FT cells with HIV-1 Gag-Pol, Rev, RDpro and vector, and to transient transfection of STAR-RDpro cells with vector alone. Clone F was least effective and not used for further development (Figure 2c).

### Establishment of continuous vector producer cells

WinPac-RD cells constitutively expressed all packaging functions, Gag-Pol, Rev and envelope, to package HIV-1 vector genomes. The final step to establish continuous LV producer cells was to express vector genomic RNA. SIN LVs contain a deletion within the LTR U3 region. This deletion makes them less genotoxic^34^,^35^,^36^ and more likely to be widely used for clinical applications. Therefore, we transfected a GFP-expressing SIN LV, SIN-pHV (Supplementary Figure S4b), into WinPac-RD cells. We aimed to produce culture supernatants with LV transduction titers of at least 10^5^ transduction units (TU)/ml, as the final product for clinical use after vector concentration and purification may be required to be of more than 10^7^ TU/ml.

**Figure 3:**
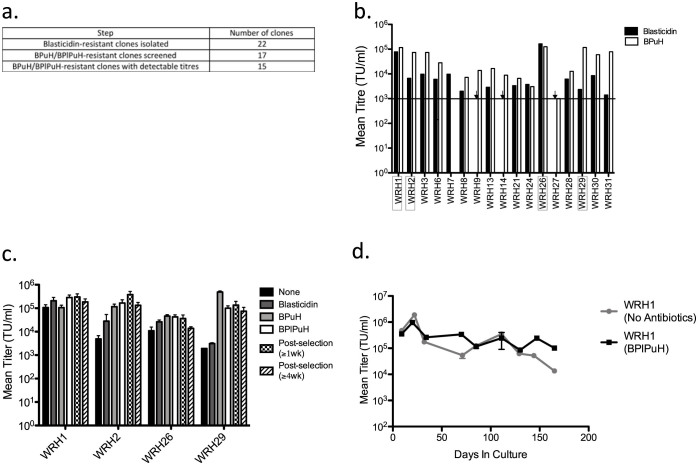
Establishment of stable producers for a GFP-coding SIN-HIV vector. (a) Screening steps for high-titer vector producer cell lines. (b) Screening of blasticidin-resistant clones stably co-transfected with SIN pHV and pSELECT Blasti MCS before and after re-selection with puromycin and hygromycin in the presence of blasticidin (BPuH). Clones WinPac-RD-HV (WRH) 1, 2, 26, and 29 were chosen for further experiments (boxed). Black horizontal line: threshold level of detection. Downward arrows: titers below threshold. Data shown represents mean of two replicates. The median titer of all screened BPuH-resistant clones was 1.66 × 10^4^ 293T TU/ml (range: 3.05 × 10^3^–1.25 × 10^5^). (c) Titers of the four WRH clones during step-wise drug re-selection (blasticin + phleomycin + puromycin + hygromycin (BPlPuH)), and after ≥4 weeks following the removal of antibiotics (mean +/− SE of two replicates). (d) Stability of titers during long-term (>5 months) culture in the presence or absence of antibiotic selection (mean +/− SE of two replicates).

Before transfection of the SIN LV, WinPac-RD cells were cultured in the presence of puromycin, hygromycin and phleomycin for 9 days to ensure the majority of cells express all the packaging functions that are genetically linked to respective antibiotic resistance genes. Subsequently, SIN pHV was co-transfected with pSELECT Blasti MCS (encoding the blasticidin resistance gene - Bsr) at a molar ratio of 10:1. Twenty two cell clones (WinPac-RD-HV shortened to WRH) were obtained from a blasticidin-resistant bulk population of cells which produced SIN-pHV at titers of >10^4^ 293T TU/ml (Figure 3a). One of these clones (WRH26) produced LV titers higher than 1 × 10^5^ TU/ml (Figure 3b, blasticidin selected). Because the stable transfection of a new vector component may be associated with a reduction in expression of pre-existing packaging functions, the clones were also re-selected with puromycin and hygromycin to ensure that the packaging components were expressed at relatively high levels. Three more clones (WRH1, WRH2, and WRH29) with titers of ≈1 × 10^5^ TU/ml were identified (Figure 3b, BPuH selected).

To confirm that re-selection with antibiotics can reproducibly increase LV titers, unselected WRH clones were thawed out and re-selected in a stepwise manner with the four antibiotics (blasticidin, puromycin, hygromycin, and phleomycin). Functional titres were determined after the addition of each of the antibiotics (Figure 3c). Importantly, selection of WRH clones with antibiotics raised titers to ≈10^5^ 293T TU/ml. Moreover, these titers were stable for ≥4 weeks of culture following removal of antibiotics (Figure 3c). To further demonstrate the stability of WinPac-derived producer cells, clone WRH1 was kept in culture with or without antibiotics and transduction titers were determined at 2–4 week intervals. Titers were relatively stable over a period of around 4 months in the absence of antibiotics, and for around 5 months in the presence of antibiotics (Figure 3d). It should be noted that the all titers shown in Figure 3 represent sub-optimal values as vectors were harvested from small numbers of cells in order to screen multiple clones and to test a number of antibiotic selection procedures.

### Harvest and processing of vectors

**Figure 4:**
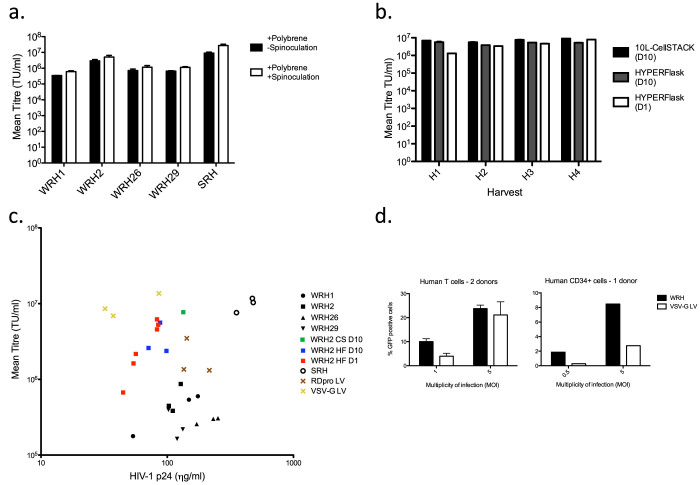
Optimization of vector production and primary cell transduction. (a) Optimised GFP infectious titers of the four WinPac-RD-HV (WRH) clones cultured in 6-well plates after re-selection with Blasticidin, Phleomycin, Puromycin, and Hygromycin (BPlPuH). Vectors were titrated on 293T cells with/without spinoculation (mean +/− SD of three independent experiments). (b) Scaled up vector harvests using 10-layer CellSTACK and HYPERFlask, collected daily from 72 h (H1) to 144 h (H4) after cell seeding, were titrated for GFP transduction. Vectors were harvested with either 10% (D10) or 1% (D1) FCS. Cells in HYPERFlask with 1% FCS lasted two more days and kept producing 5 × 10^6^ TU/ml titers (data not shown). Data shown represents mean +/− SE of two replicates. (c) GFP infectious titers and HIV-1 p24 concentration (ng/ml) of vector harvests. Data shown represents three independent harvests from each of the following: WRH1 and WRH2 (WinPac-RD derived producer clones expressing SIN HV vector harvested from T175 flasks); SRH (STAR-RDpro derived producer cells expressing non-SIN HV vector harvested from T175 flasks), RDpro LV (produced by 3 plasmid transient transfection of 293FT cells in 10 cm^2^ plates using a RDpro env expressing plasmid); VSV-G LV (produced by 3 plasmid transient transfection of 293FT cells in 10 cm^2^ plates using a VSV-G expressing plasmid). WRH2 HF D10 and WRH2 HF D1 samples represent multiple harvests obtained from clone WRH2 cultured in a HYPERFlask and harvested in the presence of 10% or 1% FBS, respectively. WRH2 CS D10 sample was obtained from clone WRH2 cultured in a 10-layer CellSTACK. (d) T cells from two donors and CD34+ cells from one donor were challenged by LV containing the GFP encoding SIN-HV vectors either stably produced by WinPac-RD-derived cells (WRH) or transiently produced by 293FT cells using VSV-G expressin plasmid (VSV-G LV) at two different vector doses: multiplicity of infection (MOI) was based on infectious titers on 293T cells. For T cells, data shown represents mean +/− SE from two donors calculated from triplicates.

A series of experiments were then conducted to determine the optimal harvesting conditions for WRH clones (data not shown). These experiments showed that higher titers were achieved when cells were fully confluent at the start of a 24-hour vector production period. Optimal harvests were collected beginning at 72 hours after seeding under following conditions: seeding density = 2.1–2.3 × 10^5^ cells/cm^2^ and harvest volume (ml): surface area (cm^2^) ratio ≈0.1. Optimized harvests from the producer clones had average titers up to 3 × 10^6^ 293T TU/ml (Figure 4a, -spinoculation). It has been previously shown that spinoculation improves the efficiency of transduction of HIV-1-based vectors by depositing the vectors onto the target cells^37^. Accordingly, spinoculation resulted in 2–3 fold increase in titers (up to 5 × 10^6^ TU/ml) (Figure 4a, +spinoculation). Vector production was also scaled up to allow the collection of 640 ml or 560 ml per harvest using 10-layer CellSTACKs (Corning) or HYPERFlasks (Corning) respectively. After scale up, vector titers had a mean of 7.28 × 10^6^ (SD = 1.47 × 10^6^) and 5.00 × 10^6^ (SD = 8.02 × 10^5^) 293T TU/ml over four days, respectively (Figure 4b). The mean productivity per cell was 1.75 (SD = 0.28) TU/cell/day. Additionally, cells tolerated reduction of FCS concentration down to 1% in HYPERFlask and continued to produce >5 × 10^6^ TU/ml up to the 6^th^ harvest (Figure 4B and data not shown). Such reduction of serum in vector harvests would help downstream vector processing.

HIV-1 p24 level and transduction titers were compared for vector harvests from stable WinPac- and STAR-derived vector producer cells as well as transient 293FT producers for RDpro and VSV pseudotyped vectors (Figure 4c). HIV p24 levels and transduction were not significantly different between RDpro pseudotyped vectors, regardless of whether they were stably or transiently produced. In contrast VSV-G pseudotyped vectors had significantly higher transduction rate per physical particle as measured by p24 ELISA (Supplementary Figure S1). Higher transduction efficiency of VSV-G LVs in comparison to RDpro-pseudotyped LVs on immortalized cell lines, such as 293T cells, have been previously reported^23^,^38^,^39^. However, of note, HYPERFlask harvests of WinPac–RD supernatants had about 10-fold higher ratio of 293T transducing units /p24 level compared with small-scale routine harvests (mean ratios of 4.2 × 10^4^ versus 3.0 × 10^3^ 293T TU/ng p24 respectively, Supplementary Figure S1).

Vector processing including concentration and purification is required before any clinical application. Because LVs pseudotyped with γ-retroviral envelopes may be mechanically more fragile than VSV-G pseudotyped vectors, we tested two concentration methods on our stably produced RDpro enveloped vectors: low-speed centrifugation at 4000 × g for 18 hr and tangential flow filtration (TFF) using 500 kDa cut-off hollow fiber (Supplementary Figure S2). The vector titers reached 7–9 × 10^7^ TU/ml in these concentration trials.

CD34+ cell and T cells are important targets in human gene therapy^8^,^9^,^10^,^11^,^12^,^13^,^14^. We therefore tested the transduction efficiency of our stably produced RDpro-pseudotyped LVs and compared it to that of transiently-produced VSV-G-pseudotyped LVs. Equal number of 293T TU were used to transduce either human bone marrow-derived CD34+ cells or human T cells by pre-loading retronectin-coated plates. For both cell types, stably produced RDpro pseudotypes outperformed VSV-G pseudotypes especially at lower MOIs (Fig. 4d).

### Effects of levels of vector components on vector titers

**Figure 5:**
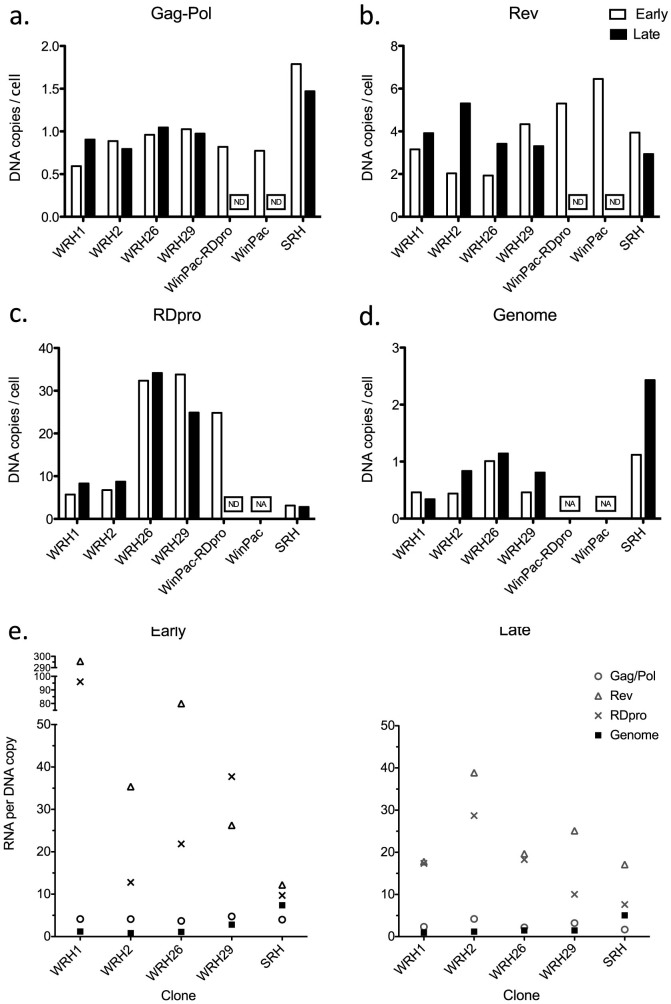
DNA copy numbers and RNA expression levels of each vector component in packaging and producer cell lines. DNA copy numbers per cell for Gag-Pol (a) Rev (b), RDpro (c) and Genome (d, primers used amplify the HIV-1 leader sequence) measured by qPCR. Cell number per reaction was estimated by performing qPCR for β-actin in parallel. Data shown represents mean of two replicates. (e) RNA expression levels per DNA copy number of each vector component are shown. RNA expression levels were measured by q-RT-PCR and normalized to the β-actin RNA expression levels in each sample and divided by the corresponding DNA copy number. Early (after ≈2–4 weeks in culture) and late (after ≈12–14 weeks in culture) time points were around 8–10 weeks apart.

**Figure 6:**
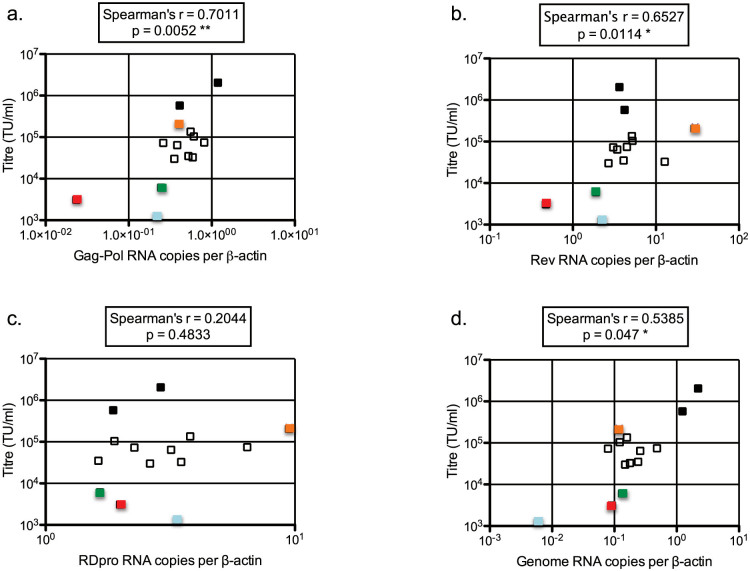
Correlation between RNA expression levels of vector components and functional titers. Points in each graph represent the RNA expression levels of the various components at the Early and Late time points for the four WinPac-RD-HV (WRH) producer clones (while growing in the presence or absence of selection antibiotics) and STAR-RDpro-HV (SRH) cells plotted against the titers determined at the time of RNA extraction. The data represents the mean of two replicates. Black: SRH, Orange: WRH1 Early BPlPuH, Red: WRH2 Early no antibiotics, Blue: WRH1 Late no antibiotics, Green: WRH26 Early no antibiotics, White: remaining WRH.

In order to determine which vector component(s) influence vector production efficiency, i.e. vector titers, we measured DNA (Figure 5) and RNA (Figure 6) levels in a number of vector producer clones at two time points during continuous culture approximately 10 weeks apart (early and late). Vector titers of corresponding harvests were also measured at the same two time points. The DNA copy number for Gag-Pol, at the lox-P tagged locus, was stable in WinPac derived cell lines (Figure 5a). Consistent with our previous report^23^, STAR-derived cells contained multiple Gag-Pol copies. Notably, the stable DNA copy numbers of the SIN-HV genome DNA in the tested clones (Figure 5d) suggest the absence of significant auto-transduction likely due to the interference phenomenon^40^. Gag-Pol RNA levels were relatively stable suggesting that the lox-P tagged locus can support high expression levels long-term (Figure 5e). RDpro env and Rev RNA levels decreased with time in some of the clones tested.

We then compared vector titers with component RNA levels to examine which RNA might be limiting for vector titer in a variety of WRH and SRH clones. Figure 6 shows that RNA levels for Gag-Pol, Rev and SIN-HV genome, but not that for RDpro env, positively correlated with transduction titers (Figure 6). This suggests that particular attention should be paid to expression levels of Gag-Pol, Rev and vector genome. Examination of individual clones suggested that any of these could limit vector titer (WRH1 (early, BPlPuH): vector genome, WRH2 (early, no antibiotics): Gag-Pol/Rev, WRH26 (early, no antibiotics): Rev, Figure 6).

### Safety characteristics

APOBEC3G (A3G) belongs to the apolipoprotein B mRNA editing enzyme catalytic polypeptide-like (APOBEC) family of proteins and was initially identified as a potent restriction factor against HIV-1 infection in human CD4+ T cells^41^. APOBEC3G-mediated G to A hypermutation in integrated proviral copies of γ-retroviral vectors produced by the HT1080-derived FLYA13 packaging cell lines has been previously demonstrated^42^. These mutations can have important consequences if they occur in the region coding for the therapeutic gene of interest, as they may lead to decreased levels of production, or the production of an inactive or immunogenic variant of the therapeutic protein. However, no hypermutation of vectors produced by 293 cells was detectable^42^. This is consistent with a previous report demonstrating that A3G RNA was undetectable in 293T cells^43^. Thus, we tested whether WinPac cells express A3G or not at various stages of their development. As expected, APOBEC3G protein was not detected by western blot in WinPac, WinPac-RD, and WinPac-RD-HV1 cells (Supplementary Figure S3a).

We hypothesized that stable LV production yields preparations containing less plasmid DNA and cell-derived contaminants, compared to transient production methods. Importantly, plasmid DNA contaminants in clinical vector preparations can potentially induce immune responses via Toll-Like Receptors^15^. To compare the relative amounts of such contaminants in untreated stably- and transiently-produced vector preparations, Q-PCR-based assays were used to detect cell-derived DNA encoding SV40T Ag and plasmid DNA (Supplementary Figure S3b). Although there were similar levels of cell-derived DNA in all preparations tested, there were higher levels of plasmid DNA in transiently produced vector preparations.

## Discussion

Here, we have demonstrated that it is possible to express HIV-1 Gag-Pol constitutively, at a high level, from a single copy cassette inserted into the producer cell genome by Cre-mediated cassette exchange. We chose to insert the target LoxP sites using a GRV vector as we previously demonstrated that GRV insertion sites would support HIV-1 Gag-Pol expression. This method could be adapted to use CRISPR-Cas technology to insert LoxP sites into known loci. It will also be possible to modify the Gag-Pol expression cassette to test other enhancer/promoters. We chose the CMV immediate early promoter since it worked well in STAR cells. Likewise, the Rev expression cassette could be modified. However, our experience shows that both Gag-Pol and Rev should be re-selectable by antibiotic resistance, and should be expressed as highly as possible as we have shown that their expression levels correlate positively with LV titer.

Furthermore, we have shown that linking expression of the various vector components with that of selectable markers ensures high titers are achieved. Practically, we would recommend re-selection with antibiotics before and after the expression of new vector component in packaging cells as well as after thawing out producer cells. Subsequently, cell culture can be scaled up and vector batch production can be reliably undertaken in the absence of antibiotics.

We chose to stably express the RDpro envelope protein to exemplify the method. Other non-cytotoxic viral envelopes could be substituted, such as those from amphotropic murine leukemia virus or Gibbon ape leukemia virus, which have been used in clinical gene therapy trials with GRV^44^. It would also be possible to use an inducible construct for a cytotoxic envelope such as VSV-G, in cells containing all the other LV components. This induction in the presence of optimal expression of Gag-Pol, Rev and SIN vector, should be more efficient than simultaneous induction of multiple components.

Our novel, clinical-grade WinPac cells with the RD114-derived envelope, RDpro, can continuously produce third generation SIN LV at titers in the order of 10^6^ TU/ml. In current successful, gene therapy trials, roughly 1–40 × 10^9^ infectious units of vectors per patient are required^8^,^9^,^10^,^11^. It is certainly feasible to produce clinically useful batches of therapeutic LV by optimized scaling-up of cell culture, vector harvest and processing using WinPac-RD packaging cells. Such continuous LV production methods will have considerable advantages over current transient vector production methods, being cheaper, more reproducible and lower in contaminants.

Compared to currently available PCLs, WinPac cells can support the production of SIN LV at superior titers compared to the other constitutive LV PCL reported to date^27^. In contrast to inducible PCLs proposed for clinical LV production, like the GPRG cell line^16^, continuous production using WinPac cells is easier to scale up and avoids the rapid decline in titers following induction. Interestingly, WinPac-derived producers had titers similar to GPRG-derived producers obtained following plasmid transfection as opposed to concatemeric array transfection, which is difficult to reproduce and is less stable.

Notably, the expression level of the SIN vector genome in our model producer cells is suboptimal and limits titers. The highest titer producer cell lines from STAR-RDpro contained a transfer vector carrying a full length LTR in the presence of HIV-1 tat. This might account for the higher expression of the vector genome RNA. Therefore, we are currently optimizing the expression level for therapeutic SIN vector production in WinPac cells using alternative techniques including RMCE at a pre-defined locus. This strategy would facilitate the reproducible construction of various producer cell lines from a master packaging cell line. Moreover, we are developing stable producer cell lines for therapeutic LV production for the treatment of X-linked Severe Combined Immunodeficiency (SCID-X1) and B cell malignancies. To facilitate the use of these cell lines, optimization of the downstream processing protocols for RDpro-pseudotyped LVs is warranted.

## Methods

### Cell Culture

HEK293T, HEK293FT (Genethon, Evry, France), HeLa, HT1080, WinPac, STAR, and FLY cells were cultured in DMEM (Dulbecco's Modified Eagle Medium) containing Glutamax (GIBCO, Carslbad, CA), supplemented with 50 U/ml Penicillin, 50 μg/ml Streptomycin (GIBCO) and 10% FBS (Sigma-Aldrich, St Louis, MO/GIBCO) at 37°C and 5% CO_2_. When indicated, antibiotics were added to the culture medium (Antibiotics and their working concentration are listed in the Supplementary Table S4). The lot numbers of all reagents added to 293FT cells and all cell lines derived from it potentially for future clinical use have been documented.

### LV production from producer cell lines

Cells were seeded at a density of 2.1–2.3 × 10^5^ cell/cm^2^. After 72 hours, cells were washed with medium and 0.08–0.1 ml/cm^2^ of medium was replaced except for HYPERFlasks for which 0.33 ml/cm^2^ medium was used. 24 hours later, vector-containing medium (VCM) was collected, passed through 0.45 μm filter and stored at −80°C. Fresh medium was added to the cells for collection after 24 hours. This process was repeated for up to six times.

### Transient LV Production

Three-plasmid co-transfection into HEK293FT cells was used to make pseudotyped LV as described previously^45^. Briefly, 6 × 10^6^ 293FT cells were seeded in 10 cm^2^ plates. 24 hours later, they were transfected using fugene6 (Promega, Madison, WI) with following plasmids: SIN pHV (vector plasmid), p8.91 (Gag-Pol expression plasmid^45^,), and either pMD.G (VSV-G env expression plasmid^46^,) or pRDproLF (RD114-derived env expression plasmid^23^,). Medium was changed after 24 hours and then VCM was collected over 24-hour periods for 3 days. Following collection, VCM was passed through 0.45 μm filter and stored at −80°C.

### LV Concentration

WinPac-RD-HV, RDpro LV and VSV-G LV preparations were concentrated by centrifugation in a Heraeus Megafuge (Thermo Scientific, Waltham, MA) at 4000 × g for18 hours at 4°C. The pellet was resuspended in ice cold X-VIVO10 (Lonza) and stored at −80°C. Alternatively, vector preparations were concentrated by tangential flow filtration (TFF) using a KrosFlo Research IIi System and a 115 cm^2^ 500 kDa cut-off PES hollow fibre (Spectrum Labs, Rancho Dominguez, CA).

### LV Titration

The functional titer of each vector preparation was determined by flow cytometric analysis for GFP expression following transduction of 293T cells. Briefly, 6 × 105 293T cells were infected with LV plus 8 μg/ml polybrene (Sigma-Aldrich) for 24 hours. Infected cells were detected by eGFP expression using FACSCalibur (BD Biosciences, San Jose, CA) and Flowjo software at 48 hours following the start of transduction. Titers were calculated from virus dilutions where 1–20% of the cell population was EGFP-positive using the following formula:
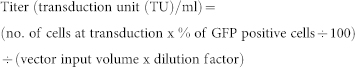


### Primary cell transduction

For T cell transduction, whole blood was collected from donors following signed consent under sterile conditions. PBMCs were isolated by Ficoll (GE Healthcare, Little Chalfont, UK) gradient centrifugation, re-suspended in X-VIVO 10 (Lonza, Basel, Switzerland), stimulated overnight with 0.5 μg/ml OKT3 (anti-CD3, Miltenyi Biotech, Auburn, CA) and 0.5 μg/ml anti-CD28 (Miltenyi Biotech). IL-2 (Proleukin, Chiron, Emeryville, CA) was added at a concentration of 100 international units (IU)/ml following overnight stimulation. On the next day, T-cells were harvested, seeded at 3 × 10^5^ cells per well, and spun at 1000 g for 40 minutes at room temperature on 24-well plates previously coated with the CH-296 fragment of fibronectin (Retronectin, Takara, New York, NY) and preloaded with TFF-concentrated vector supernatant at MOI 1 or 5 (based on 293T transduction units). After 72 hours incubation, T cells were harvested and re-suspended in fresh X-VIVO 10 medium supplemented with 100 TU/ml IL-2. 72 hours later (6 days post-transduction), transduced T cells were analyzed for GFP expression by flow cytometry (FACSCalibur, BD Biosciences).

Human CD34+ cells were isolated from G-CSF mobilized peripheral blood of a healthy donor using the Diamond CD34 Isolation Kit (Miltenyi Biotec). Cells were cultured in X-VIVO 10 plus 1% human serum albumin (HSA), supplemented with stem cell factor (hSCF) at 100 ng/ml; human Flt3-ligand (hFlt-3L) at 100 ng/ml; thrombopoietin (hTPO) at 100 ng/ml; and human interleukin 3 (hIL3) at 20 ng/ml (all from Peprotech, London, UK) for three days before transduction.

For CD34+ cell transduction, 24-well plates were coated with Retronectin (Takara). Concentrated GFP-encoding LVs (WinPac-RD-HV1 or VSV-G LV) were preloaded (by centrifugation at 1200 g at 32°C for 40 minutes) onto the retronectin-coated plates and the supernatant was discarded. 1 × 10^5^ hCD34+ cells were transduced overnight at MOI 0.5 or 5 (based on 293T transduction units). 96 hours later, transduced CD34+ cells were analyzed for GFP expression by flow cytometry (FACSCalibur, BD Biosciences).

### Quantitative PCR (Q-PCR)

To determine DNA copy number per cell of a construct encoding a vector component (e.g. Gag-Pol cassette) in packaging/producer cells, SYBR green-based Q-PCR was used. Initially, genomic DNA (gDNA) was extracted from 2 × 10^6^ cells using DNeasy Blood and Tissue Kit (Qiagen, Crawley, UK) following the manufacturer's instructions. gDNA concentration was determined by spectrophotometry and adjusted to 50 ng/μl. 100 ng of gDNA was used as template for Q-PCR reactions using the QuantiTect SYBR Green PCR Kit (Qiagen) and ABI 7500 Real-Time PCR system (Applied Biosystems, Warrington, UK). PCR reactions were performed at 95°C for 15 min, followed by 40 cycles of 95°C for 15 s, 55°C for 30 s, 72 for 30 s. A melting curve was run following each assay. All Q-PCR reactions were performed in duplicates.

The standards and primers used including primer sequences are summarized in Supplementary Table S2. For Gag-Pol, primers Q-gagpol-F and Q-gagpol-R were designed to anneal at the frameshift region between *gag* and *pol* genes, which was identical in sequence in all the HIV-1 Gag-Pol constructs used in this work. For vector genome, primers GT248 and GT249, which anneal to and amplify the HIV-1 leader region were used. For Rev, primers Q-Rev-F and Q-Rev-R were used, and for RDpro, primers Q-RD-F and Q-RD-R were used. Standards used in all QPCRs were 10^5^, 10^4^, 10^3^, 10^2^, and 10^1^ plasmids/reaction. Standards used were as follows: p8.91 for Gag-Pol and Rev; pHV for HIV-1 leader region; for β actin and RDpro, the standards were made by cloning the PCR product from HB-actin-F and RC or Q-RD-F and RC respectively, into pGEM T easy (Promega).

To calculate the DNA copy number per cell, β actin was quantified in parallel to any gene of interest and divided by 6 to give the number of cells per reaction. This was done assuming 293FT cells are triploid, and that the primer pair used (HB-actin-F and HB-actin-RC) detect the β-actin gene (on Chromosome 7) and β-actin pseudogene (on Chromosome 11).

### Reverse transcription quantitative PCR (RT-Q-PCR)

To analyze gene expression of a vector component (e.g. Gag-Pol cassette) in packaging/producer cells, SYBR green-based Q-PCR was used. Initially, RNA was extracted from 2–4 × 10^6^ cells using RNeasy Mini Kit (Qiagen) following the manufacturer's instructions. RNA was quantified by spectrophotometry and concentration was adjusted to 100 ng/μl. cDNA was prepared using the Quantitect Reverse Transcriptase kit (Qiagen) following the manufacturer's instructions. 100 ng of the synthesized cDNA was used as template for Q-PCR using the QuantiTect SYBR Green PCR Kit (Qiagen) and ABI 7500 Real-Time PCR system (Applied Biosystems). Q-PCR reactions were performed at 95°C for 15 min, followed by 40 cycles of 95°C for 15 seconds, 55°C for 30 seconds, 72 for 30 seconds. A melting curve was run following each assay. All Q-PCR reactions were performed in duplicates. RNA copy number for each gene was normalized to β-actin (endogenous control gene) RNA copy number, which was determined in parallel for each reaction. The standards used in each RT-Q-PCR assay were 10^7^, 10^6^, 10^5^, 10^4^, and 10^3^ plasmids/reaction. The primers and standards used were identical to those used for Q-PCR (Supplementary Table S2).

### Q-PCR-based assay for detection of cell-derived and plasmid DNA contaminants

Detection of cell-derived SV40 TAg-encoding DNA^47^ and plasmid DNA^48^ was done using previously reported primer pairs. PCR reactions were prepared and conducted as detailed above using 2 μl of untreated VCM as a template per reaction. Reactions were performed at 95°C for 15 min, followed by 40 cycles of 95°C for 15 seconds, 57°C (SV40 TAg) or 60°C (AmpR) for 30 seconds, 72°C for 60 seconds. A melting curve was run following each assay. Q-PCR reactions were performed in triplicates. Details of primers and standards used for each target are listed in Supplementary Table S3.

### Western Blot

To prepare cell lysates, 2–3 × 10^6^ cells were washed with ice-cold PBS and lysed using 1% Triton-X100 in PBS-T (0.1% Tween20 in PBS) in the presence of 1× protease inhibitor. Following 10–15 min incubation on ice, whole cell lysates were clarified by centrifugation at 13,000 RPM, 4°C for 20 min. Total protein concentration was measured using the Pierce BCA Protein Assay Kit (Thermo Scientific). An equal amount of protein (20–25 μg) from each sample was mixed with Laemmli Buffer, heated at 90°c for 5 min. Samples were separated by electrophoresis on 10% SDS-polyacrylamide gel at 120 v for 2 hours, and then electrotransferred at 40 v for 1.5 hour onto Hybond ECL nitrocellulose membrane (GE Healthcare). The membranes were blocked with 5% skimmed milk in PBS-T (blocking buffer) for 1 hour, and then incubated with the primary antibody (diluted in blocking buffer) overnight at 4°C. After that, the membranes were washed with PBS-T for 5 min five times, incubated with the HRP-conjugated secondary antibody (diluted in blocking buffer) for 1 hour at room temperature, washed as before, and then incubated with LimiGLO chemiluminescent substrate (Cell Signaling Technology, Beverly, MA) at room temperature for 1 minute. Lastly, the membranes were exposed to Hyperfilm ECL (GE Healthcare) for signal detection. For detection of APOBEC3G, polyclonal rabbit anti-APOBEC3G (kind gift from Michael Malim, King's College London) was used as primary antibody, and polyclonal swine anti-rabbit IgG (P0399, DAKO, Glostrup, Denmark) was used as secondary antibody. For detection of α-tubulin, mouse anti-α-tubulin (T6199, Sigma-Aldrich) was used as primary antibody, and polyclonal rabbit anti-mouse IgG (P0260, DAKO) was used as secondary antibody.

### HIV-1 p24 ELISA

To estimate physical LV production, HIV-1 p24 concentration in serially diluted VCM was determined using the Lenti-X p24 Rapid Titer Kit (Clonetech, Mountain View, CA) following the manufacturer's instructions.

## Author Contributions

K.S.S., M.K.C. and Y.T. wrote the paper. M.K.C., J.M., S.L.S. and Y.T. designed WinPac constructs. S.L.S., D.E. and R.B. produced the data in Figure 1b and c. G.S. and A.T. produced Clone F cells. S.B.K. and K.S.S. produced the data in Figures 2 and 3. K.S.S. performed experiments in Figures 4–6.

## Supplementary Material

Supplementary Information SUPPLEMENTARY MATERIALS
