# Supplementary material for: Construction of stable packaging cell lines for clinical lentiviral vector production
Source: Sci Rep. 2015 Mar 12;5:9021. doi: 10.1038/srep09021 (PMC4356972; doi:10.1038/srep09021)
Supplement: Supplementary Information — SUPPLEMENTARY MATERIALS [file srep09021-s1.pdf]

SUPPLEMENTARY MATERIALS, Sanber et al.

Title: Construction of stable packaging cell lines for clinical lentiviral vector production

Khaled S Sanber*,[1,3], Sean B Knight*,[1], Sam L Stephen[1], Ranbir Bailey[1], David Escors[1], Jeremy Minshull[4], Giorgia Santilli[2], Adrian Thrasher[2], Mary K Collins,[1,3] and Yasuhiro Takeuchi**,[1]**

[1] Division of Infection and Immunity/[2] Institute of Child Health, University College London, London, UK
[3] National Institute for Biological Standards and Control, South Mimms, UK
[4] DNA2.0, Menlo Park, CA94025, USA

***KS/SK, equal contribution**
****MC/YT, equal contribution**

<u>Contents.</u>

Supplementary Tables S1 – S3
Supplementary Figures S1 – S3
Supplementary Note ‘Construction of packaging cell lines’ including Table S4 and Figures S4 – S6

Table S1. Codon Adaptation Indices of HIV-1 Gag and Pol Genes

HIV-1 Gag Gene	Codon Adaptation Index
Wild Type	0.725
Codon Optimized - WinPac	0.862
Codon Optimized - STAR	0.852

HIV-1 Pol Gene	Codon Adaptation Index
Wild Type	0.703
Codon Optimized - WinPac	0.895
Codon Optimized - STAR	0.843

Table S2. Primers for (RT-)Q-PCR.

Component	Standard	Primer Name	Primer Sequence
**HIV-1 Gag/Pol**	p8.91	Q-gag/pol-F	AAGAGAGCTTCAGGTTTGGG
		Q-gag/pol-RC	TGCCAAAGAGTGATCTGAGG
**HIV-1 Rev**	p8.91	Q-rev-F	TGTGCCTCTTCAGCTACCAC
		Q-rev-RC	CAATATTTGAGGGCTTCCCA
**RDpro envelope**	pRD	Q-RD-F	AACTCCCAACAGGAATGGTC
		Q-RD-RC	TTAAGTAGGCCGTCTTGCCT
**HIV-1 leader region**	pHV	GT248	TGTGTGCCCGTCTGTTGTGT
		GT249	GAGTCCTGCGTCGAGAGAGC
**Human β-Actin**	pHB-actin	HB-actin-F	TGGACTTCGAGCAAGAGATG
		HB-actin-RC	TTAAGTAGGCCGTCTTGCCT

Table S3. Primers for safety assays.

Target Sequence	Standard	Primer Name	Primer Sequence
**SV40 TAg**	pBABE-puro SV40LT	SV40TAg F	TGAGGCTACTGCTGACTCTCAACA
		SV40TAg RC	GCATGACTCAAAAAACTTAGCAATTCTG
**AmpR**	pHV	AmpFP	ACTCGCCTTGATCGTTGGG
		AmpRP	GTTGCCATTGCTACAGGCATC

Table S4. Antibiotics used in cell culture

Antibiotic	Associated vector component	Working concentration (μg/ml)
**Puromycin (Invivogen)**	Gag-Pol	1
**Hygromycin (Invivogen)**	Rev	100
**Phleomycin (Invivogen)**	Env	30
**Blasicidin (Invitrogen)**	Genome	10

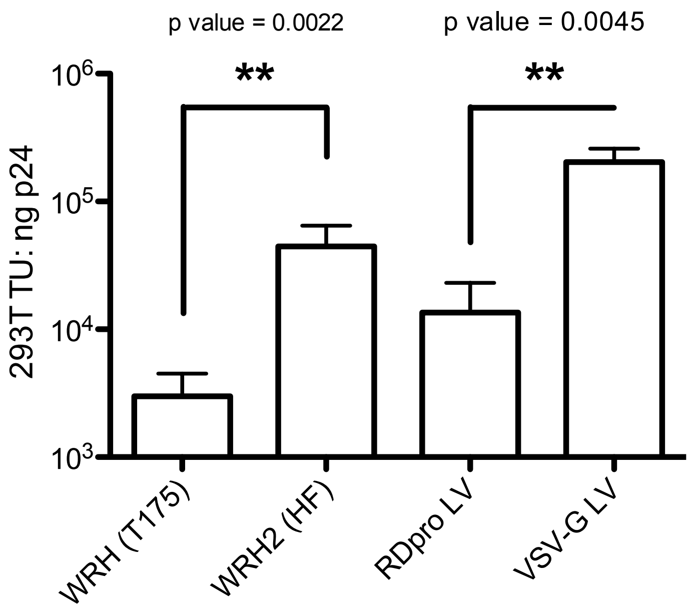

Figure S1. Infectivity of stably and transiently produced LV preparations. Ratio of 293T transducting units: p24 levels (ηg/ml) determined by ELISA was used as a measure of infectivity. WRH (T175) represents the mean ratio for four different WinPac-RD-HV clones (mean ratio for each clone was determined from 3 vector preparations obtained from T175 flasks). WRH (HF) represents the mean ratio for 9 vector preparations obtained from HYPERFlasks and produced by clone WinPac-RD-HV2. For each of RDpro LV and VSV-G LV, data shown represents the mean ratio for 3 vector preparations produced by transient transfection of 293FT cells in $10cm^2$ plates. Error bars represent SD. Unpaired t-test was used to compare mean ratios.

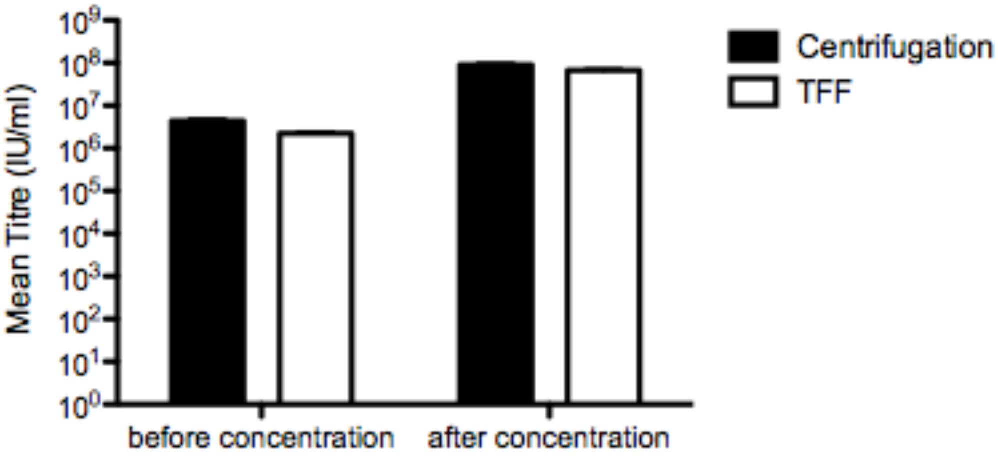

Figure S2. GFP infectious titers of WRH2-produced vectors before and after concentration by centrifugation (180-fold reduction in volume) or tangential flow filtration (TFF) (100-fold reduction in volume). Data shown represents mean titre and range of duplicate titrations.

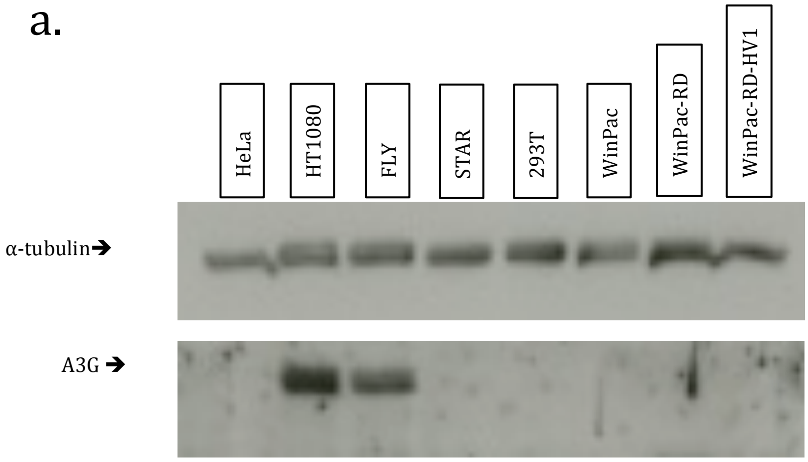

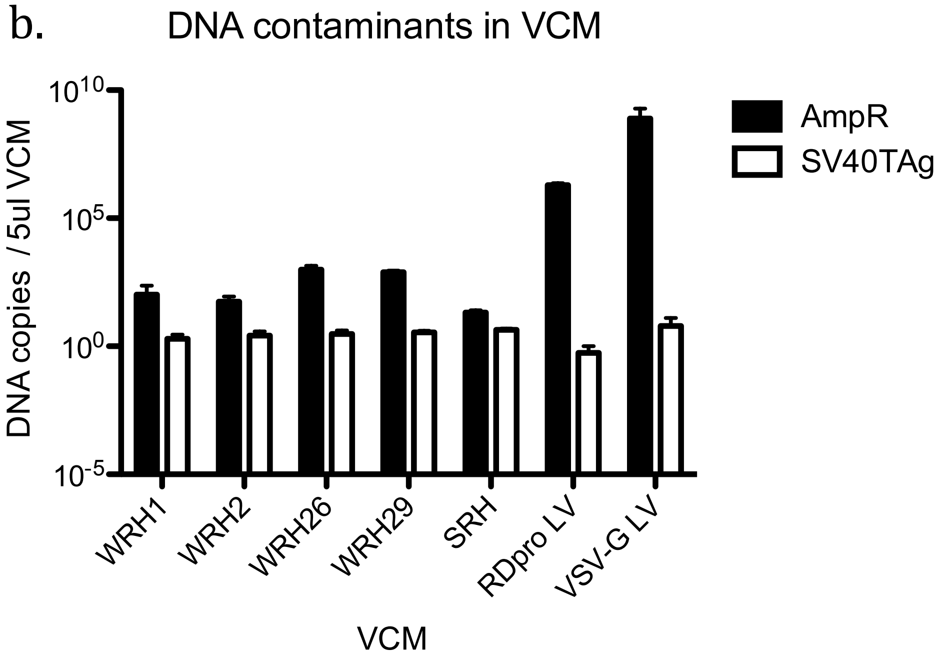

Figure S3. Safety assays. (a) Western blot for APOBEC3G on lysates derived from 293FT, WinPac, WinPac-RD and WinPac-RD-HV cells. HeLa cells were used as a negative control, while HT1080 and FLY cells (HT1080-derived γ-retroviral packaging cell line) were used as positive controls (1). (b) Detection of plasmid and cell-derived DNA in stably- and transiently-produced vectors. Equal volume of untreated culture supernatant for each vector was used in the Q-PCR assays. Primers that amplify a segment of the ampicillin resistance gene (AmpR) were used to detect DNA, mainly derived from transiently transfected plasmids. Primers that amplify a segment of the gene encoding SV40TAg were used to detect cell-derived DNA (n=3, data shown represents mean titre and SD).

Supplementary Note:
Construction of packaging cell lines

Plasmids
For introduction of double loxP sites by MLV vector transduction, pSLS51 containing an MLV vector which has a LE mutant loxP site in the 3'LTR U3 region and a CMV promoter-driven eGFP-hygro transgene, was constructed (Supplementary Figure S4a, sequence deposited in GenBank KP659661). pSLS94 (Supplementary Figure S4a, sequence deposited in GenBank KP659662) harbors a cassette coding for promoter-less puroR and CMV promoter-driven synthetic HIV gag-pol genes flanked by two RE mutant loxP sites. This cassette is to be recombined between the two mutant loxP sites created by pSLS51 vector. pCAGGS Cre expressing Cre recombinase has been described previously (2). pCEP4 Rev was constructed by inserting HIV Rev into pCEP4 Plasmid (Invitrogen, Carlsbad, CA) using the Hind III and Xho I restriction endonuclease sites. pRDproLF contains the RD114 envelope with a HIV protease cleavage site under the control of an MLV LTR, and the Phleomycin resistance gene under the control of another MLV LTR (1, 3). To construct SIN PHV (Supplementary Figure S4b), the SIN lentiviral LTR from UCOE-gamma-C (4) was cloned into pHV (3) in place of the wild type lentiviral LTR. Briefly, pHV was digested with BamHI (Promega, Madison, WI) and Apa I (New England Biolabs, Ipswich, MA). The resulting ~5.7kb fragment was kept as backbone. The ~2.2kb fragment was digested with Sac II (Promega) and a ~1.2kb fragment was obtained and kept as the first insert. The SIN LTR from UCOE-gamma-C was amplified by PCR using KOD polymerase (Novagen–EMD Millipore, Darmstadt, Germany) by primers Sac WPRE-F and ApaI UCOE RC. This PCR product was then cut with SacII (Promega) and ApaI. Finally, SIN pHV was constructed by a three-piece ligation of the ~5.7kb pHV backbone, the 1.2kb pHV fragment, and the SIN LTR from UCOE-gamma-C. pSelect-Blasti-MCS (Invivogen, Carlsbad, CA) encodes the Blasticidin resistance gene (BSr) under the control of the CMV promoter.

Tagging LoxP sites in 293FT cells.
Recombinase-mediated cassette exchange (RCME) was used to stably express HIV *gag-pol*. In principle, this involved 'tagging' a chromosomal location that was able to support robust expression of a GFP cassette and then using cre-recombinase to exchange GFP for HIV *gag-pol*. To tag a high expresser site, 293FT cells were infected at a low MOI with pSLS51 MLV vector and selected in hygromycin to obtain clones with a single copy of the vector. One of these, clone 2G had a relatively high mean fluorescence intensity (MFI) (main paper Figure 1b) that was stable over 50 passages and a single vector copy per cell by QPCR, and was thus chosen for the next stage, where GFP was exchanged for *gag-pol* by RCME. The integration site in clone 2G was cloned by inverse PCR and mapped to the X chromosome in the first intron of midline 1 gene (*MID1*) in the reverse orientation at nucleotide position X: 10619185 in human genome assembly GRCh38 (Dec 2013 release).

Expression of HIV-1 Gag-Pol by RCME.
pSLS94 containing the exchange cassette has a codon-optimised *gag-pol* with a histidine to glutamine change at amino acid 87 in HIV capsid (5, 6). To enable

selection of successful recombination events, a promoter-less puromycin resistance gene was cloned downstream of the 5' mutant loxP site. This meant that in a successful recombination, the promoter-less puromycin resistance gene would be placed downstream of the MLV U3 region of the tagging vector, and thus would be transcribed conferring resistance to puromycin. Importantly, directionality of recombination was ensured by using the mutant loxP sites as after recombination these generate a doubly-mutant loxP site and a full wild type loxP site at the tagged genomic location, which cannot recombine efficiently (see main paper Figure 1a). Co-transfection of Cre-recombinase, pCAGGS-Cre and pSLS91 led to successful recombination in several clones, which gained HIV-1 p24 and lost GFP expression (see main paper Figure 1c). The most promising clone, clone 57, was chosen for further development.

Stable transfection of HIV-1 Rev in Clone 57

pCEP4 Rev plasmid (Supplementary Figure S5a) was digested with *EcoRV* (Promega) and *NruI* (Promega). This released a 3.8kb fragment containing Rev under the control of the CMV promoter and the Hygromycin resistance gene under the control of the pTK promoter, which was extracted from an agarose gel using Gel Extraction Kit (Qiagen, Crawley, UK). A confluent plate of Clone 57 cells was passaged 1:6 into a 10cm plate the night before transfection with 1.5μg of Rev/Hygro fragment using Fugene (Roche, Penzberg, Germany) and Optimem (Gibco, Carlsbad, CA), after 48h cells were passaged 1:20 and then 5 serial 3 fold dilutions were made, each dilution was used to seed a 10cm plate in DMEM with 100μg/ml Hygromycin B (Calbiotech). Fourteen hygromycin resistant clones were expanded and all of them expressed some *rev* RNA, as measured by Q-RT-PCR (Supplelmentary Figure S5b). A subset of the 57R clones were analysed further by measuring expression of *gag-pol* by Q-RT-PCR and the titer produced after transient transfection of SIN pHV and VSV-G envelope. Most of the clones had maintained some expression of *gag-pol* (Supplementary Figure S5c). The clone expressing the highest level of *rev* RNA was 57R10, although *rev* expression decreased about 2 fold in the first 7 days in culture, i.e. between Supplementary Figure S5b and S5c. 57R10 also had the highest titer out of the 57R clones, which was about 10 fold lower than STAR cells (Supplementary Figure S5d). Although the level of rev expression in 57R10 fluctuated, it was consistently higher than two other promising clones (57R1 and 57R6) over more than 30 days in culture (Supplementary Figure S5e). The decrease in *rev* expression between days 1 and 10 in culture did not cause a substantial change in transient titer after SIN pHV and VSV-G transfection. Thus 57R10 was chosen for the next step and renamed WinPac.

Stable transfection of RDpro in WinPac cells

The envelope stably expressed in WinPac cells was a derivative of RD114 *env*, which has a HIV protease cleavage site in the cytoplasmic tail. The plasmid pRDproLF encoding RDpro contains two promoters derived from an MLV LTR, which drive expression of RDpro and the phleomycin resistance gene (Supplementary Figure S6a). pRDproLF was linearised by the restriction endonuclease enzyme Ssp I (Promega) and extracted from an agarose gel using Gel Extraction Kit (Qiagen). A confluent 10cm plate of WinPac cells was passaged 1:6 into a 10cm plate the night before transfection with 2.6μg linearized

pRDproLF plasmid using Fugene (Roche) and Optimem (Gibco). After 48h, cells were passaged 1:20 and then 5 serial three-fold dilutions were made, each dilution was used to seed a 10cm plate in DMEM with 30μg/ml Phleomycin (Invivogen). Expression of RDpro varied in the 12 phleomycin resistant clones that were expanded for analysis (Supplementary Figure S6b). Many of these clones had also lost expression of *rev* or *gag-pol* (Supplementary Figure S6c). The clone with the highest expression of RDpro had maintained expression of *gag-pol* and *rev*, and also produced the highest titer after transfection with the SIN lentiviral vector, SIN pHV, at around 10^5 infectious units per ml (Supplementary Figure S6d). The titer produced by 57R10E was not significantly different form STAR RDpro transfected with the same vector, or transient transfection of 293FT cells with the RDpro envelope, packaging components and SIN pHV. The 57R10E clone was renamed WinPac-RD and used for further study.

Pseudotyping SIN pHV with RDpro envelope decreased titer about 5 fold in comparison to VSV-G in transient transfection in 293FT cells, which was statistically significant (Supplementary Figure S6d, p=0.002, t test). This is consistent with reports of SIV lentiviral vectors pseudotyped with modified RD114 envelope glycoproteins, where the latter had about 5 fold lower titers when titrated on the human cell line TE671. However, lentiviral vectors pseudotyped with RD114 with an MLV cytoplasmic tail had a higher titer than VSV-G pseudotypes, when titrated on peripheral blood CD34+ cells (7). This result has been replicated in HIV-1 lentiviral vectors (8) and RDpro pseudotyped lentiviral vectors produced from STAR cells have also been shown to transduce CD34+ cells efficiently (9). Therefore, stably produced RDpro pseudotyped vectors with comparable titer to transient RDpro pseudotypes in our system would be likely to perform as well as transient VSV-G pseudotypes on CD34+ cells.

Stable co-transfection of SIN pHV and pSelect Blasti MCS in WinPac-RDpro cells

To stably express the SIN vector genome in WinPac-RDpro, SIN pHV was co-transfected with pSelect Blasti MCS (Invivogen), an expression plasmid containing the blasticidin resistance gene (BSr) under the control of the CMV promoter. SIN pHV was co-transfected at a 10:1 molar ratio to pSelect Blasti MCS. Briefly, cells were passaged 1:6 the day prior to transfection with 1.5μg pSelect Blasti MCS and a 10 fold molar excess of SIN pHV using Fugene (Roche) and Optimem (Gibco). After 48h cells were passaged 1:20 and then 5 serial three-fold dilutions were made, each dilution was used to seed a 10cm plate in DMEM with 10μg/ml Blasticidin S HCl (Invitrogen).

Screening clones following expression of a new vector component

Following the expression of each vector component into the (pre-) packaging cells, the isolated clones were screened for titres obtained after transiently complementing the missing vector components as well as RNA expression levels (by RT-Q-PCR). The best clones identified following this screening process was used for further experiments. Following are details of the screening assays performed:

Transient LV production from packaging cells

All virus production from packaging cells was done in 6 well plates, unless otherwise specified. On day 0 cells were seeded at between 2 x 10^5 and 1.6 x 10^6 cells per well. On day 1, cells were transfected with missing packaging components and vector. For each well, 25μl Optimem (Gibco) was added to a sterile microcentrifuge tube, and 2.25μl Fugene (Roche) added. A total of 437ng of DNA was assembled in 437μl with sterile water (Baxter) and added to the Optimem/Fugene mix, incubated at room temperature for 15min and then added dropwise to cells. pGEM T easy plasmid (Promega) was used to make up the DNA to 437ng when packaging cells were transfected with missing packaging components. When producer cells (containing all packaging components and a vector) were tested in parallel, nothing was transfected, and the medium was simply changed at days 1 and 2.

RNA expression levels of vector components in clones

Determination of RNA expression levels of vector components in packaging/producer cells was done using RT-Q-PCR as described above.

Antibiotic selection

Since all vector components are genetically linked to selectable antibiotic-resistance genes, we used the corresponding antibiotics to ensure the majority of cells express the various packaging components. This was typically done before and after the introduction of a new packaging component, and in the case of producer cells, prior to beginning a period of vector collection/harvest.

For monoclonal populations, this was done in a stepwise manner. One antibiotic was added and cells were passaged until no significant cell death was noted. At that point the next antibiotic was introduced and the same procedure was followed. Once selection is complete, up to four antibiotics can be re-introduced simultaneously, for example, following a period of antibiotic-free culture or after freeze-thaw cycles.

The antibiotics used are listed in Table S4.

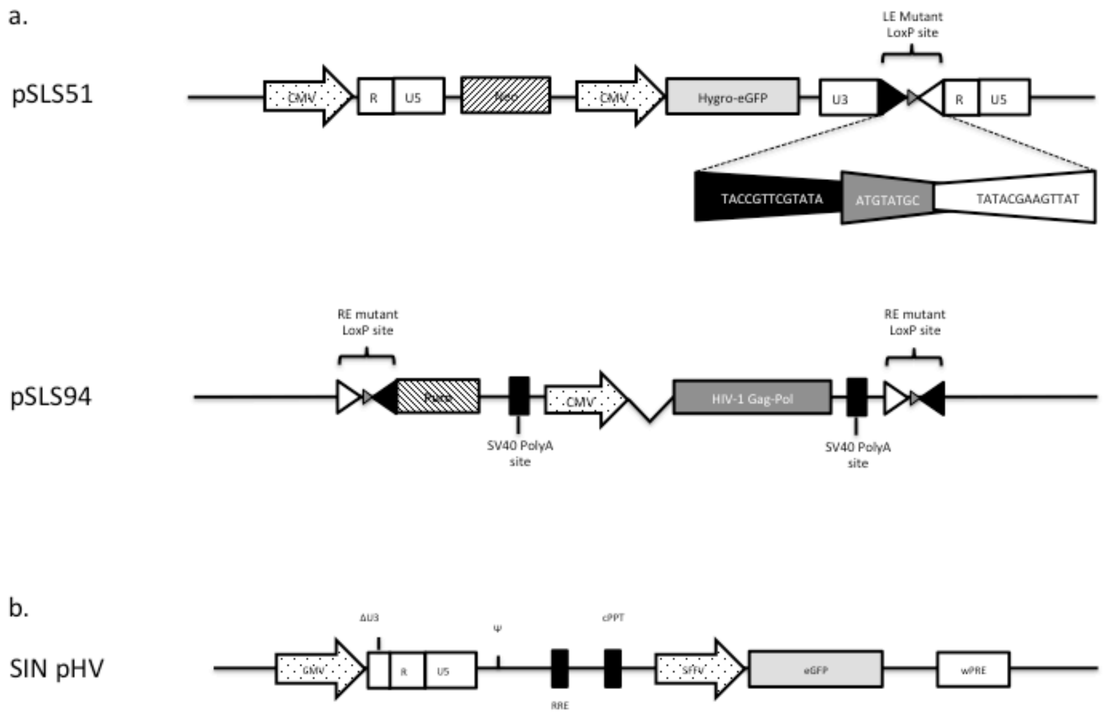

Figure S4. Schematic representation of constructs, pSLS51 and pSLS94.

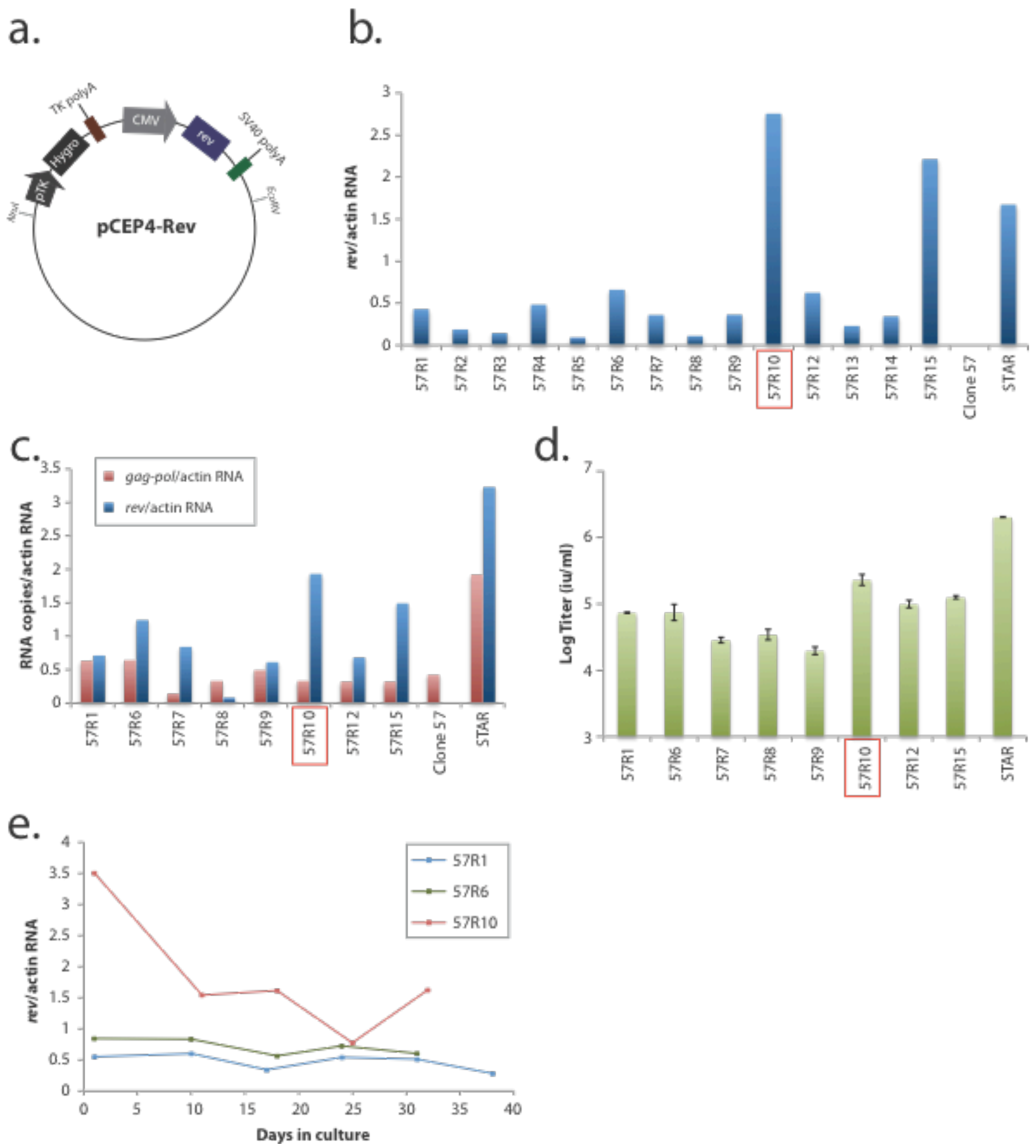

Figure S5. Stable expression of *rev* in clone 57. (a.) Plasmid map of pCEP4-Rev. pCEP4-Rev was digested with blunt cutting enzymes NruI and EcoRV, and the fragment containing the hygromycin and *rev* expression cassettes was stably transfected in clone 57 cells. pTK, Herpes simplex virus thymidine kinase promoter; Hygro, hygromycin B resistance gene; TK polyA, herpes simplex virus thymidine kinase polyA signal; CMV, human cytomegalovirus immediate-early promoter; SV40 polyA, simian virus-40 polyA signal. (b.) Expression of *rev* RNA in 14 hygromycin resistant clones (57R clones), measured by Q-RT-PCR on cDNA. This measurement was made at the time that the first liquid nitrogen stocks were made for each clone and is noted as day 0. The number of rev copies in each reaction was normalised to the number of actin copies in a parallel Q-RT-PCR. As the Q-RT-PCRs for the clones were carried out in 4 separate reactions, STAR cDNA was used as an internal control in each Q-RT-PCR. The clone chosen for progression to the next stage is enclosed in a red box. (c.) *gag-pol* and *rev* RNA expression was measured by Q-RT-PCR in a subset of 57R clones and STAR after 7 days in culture, and normalised to actin RNA expression, measured in a parallel

Q-RT-PCR. (d.) Supernatant from 57R clones and STAR was titrated on 293FT cells 48h after transient transfection of the SIN lentiviral vector, SIN pHV and the VSV-G envelope. Average log vector titers are shown from two experiments for each clone, error bars indicate standard deviation. (e.) Expression of *rev* in 3 57R clones over 30-35 days, measured by Q-RT-PCR, STAR cDNA was used as an internal control.

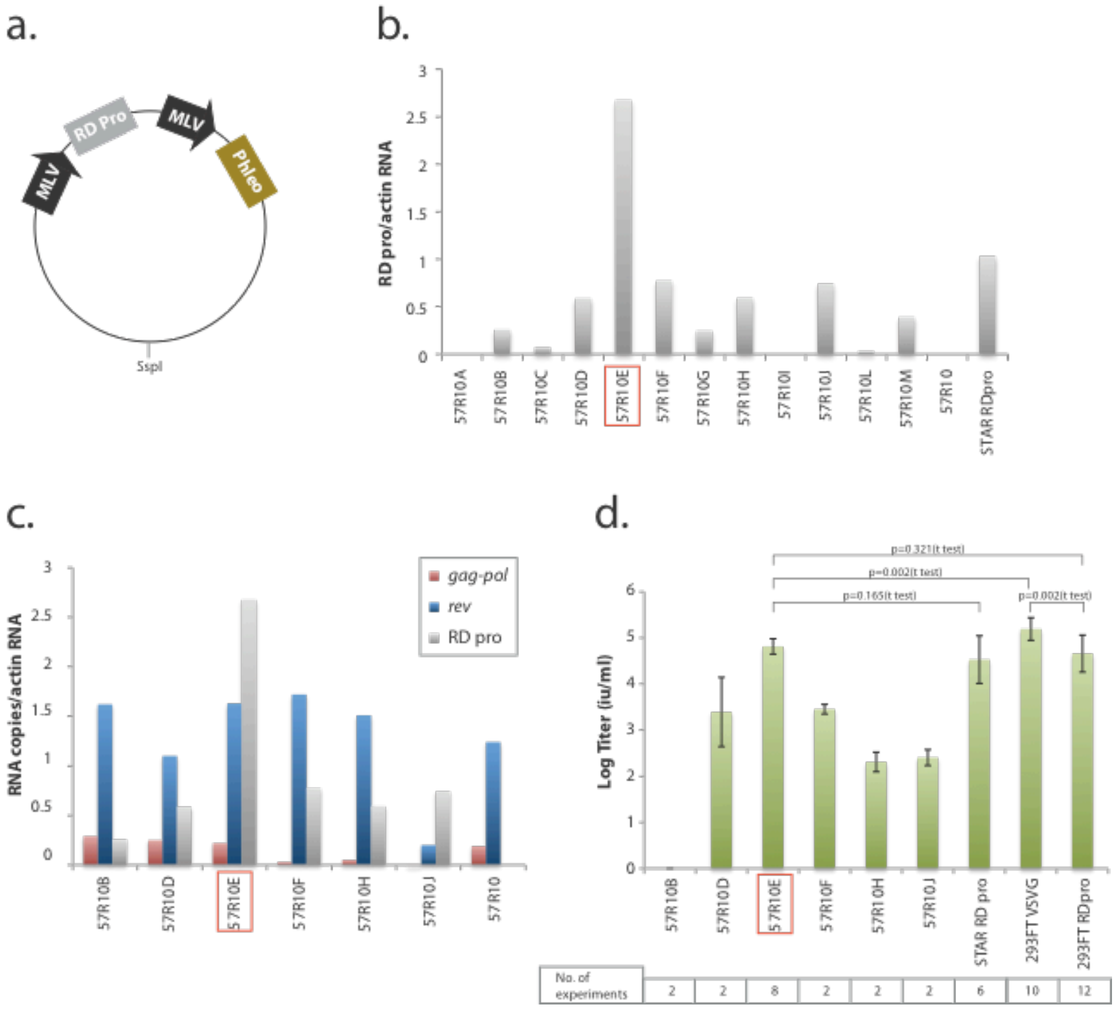

Figure S6. Stable expression of RD pro envelope in clone 57R10. (a.) plasmid map of RDpro plasmid used in stable transfection. Two MLV LTRs drive expression of RDpro gene and phleomycin resistance gene (phleo). The SspI restriction endonuclease enzyme was used to linearise the plasmid before stable transfection. (b.) Expression of RDpro envelope in 12 phleomycin resistant clones, clone 57R10 and STAR RDpro. (c.) *gag-pol* and *rev* RNA was measured in a subset of phleomycin resistant clones, by Q-RT-PCR and normalised to actin RNA copies from a parallel Q-RT-PCR. (d.) Titers of a subset of phleomycin resistant clones and STAR after transient transfection of a SIN lentiviral vector (SIN pHV). As a control, 293FT cells were transfected with p8.91 (encoding *gag-pol*, *rev* and *tat*), SIN pHV and either VSV-G envelope (293FT VSV-G) or RDpro envelope (293FT RDpro). The number of experiments is shown below each clone/cell line. Statistical tests between 57R10E and the controls are shown, only the difference between 57R10E and 293FT VSV-G was significant (p=0.002), the test that was used is shown in brackets. 293FT transfected with VSV-G gave a significantly higher titer than 293FT transiently transfected with RDpro (p=0.002). Clone 57R10E was chosen for progression to the next stage and is enclosed in a red box in (b.), (c.) and (d.).

REFERENCES

1. Cosset FL, Takeuchi Y, Battini JL, Weiss RA, Collins MK. High-titer packaging cells producing recombinant retroviruses resistant to human serum. J Virol. 1995;69(12):7430-6.
2. Araki K, Araki M, Miyazaki J, Vassalli P. Site-specific recombination of a transgene in fertilized eggs by transient expression of Cre recombinase. Proc Natl Acad Sci U S A. 1995;92(1):160-4.
3. Ikeda Y, Takeuchi Y, Martin F, Cosset FL, Mitrophanous K, Collins M. Continuous high-titer HIV-1 vector production. Nat Biotechnol. 2003;21(5):569-72.
4. Zhang F, Thornhill SI, Howe SJ, Ulaganathan M, Schambach A, Sinclair J, et al. Lentiviral vectors containing an enhancer-less ubiquitously acting chromatin opening element (UCOE) provide highly reproducible and stable transgene expression in hematopoietic cells. Blood. 2007;110(5):1448-57.
5. Ikeda Y, Ylinen LM, Kahar-Bador M, Towers GJ. Influence of gag on human immunodeficiency virus type 1 species-specific tropism. J Virol. 78. United States2004. p. 11816-22.
6. Chatterji U, Bobardt MD, Stanfield R, Ptak RG, Pallansch LA, Ward PA, et al. Naturally occurring capsid substitutions render HIV-1 cyclophilin A independent in human cells and TRIM-cyclophilin-resistant in Owl monkey cells. J Biol Chem. 280. United States2005. p. 40293-300.
7. Sandrin V, Boson B, Salmon P, Gay W, Negre D, Le Grand R, et al. Lentiviral vectors pseudotyped with a modified RD114 envelope glycoprotein show increased stability in sera and augmented transduction of primary lymphocytes and CD34+ cells derived from human and nonhuman primates. Blood. 2002;100(3):823-32.
8. Di Nunzio F, Piovani B, Cosset FL, Mavilio F, Stornaiuolo A. Transduction of human hematopoietic stem cells by lentiviral vectors pseudotyped with the RD114-TR chimeric envelope glycoprotein. Hum Gene Ther. 2007;18(9):811-20.
9. Relander T, Johansson M, Olsson K, Ikeda Y, Takeuchi Y, Collins M, et al. Gene transfer to repopulating human CD34+ cells using amphotropic-, GALV-, or RD114-pseudotyped HIV-1-based vectors from stable producer cells. Mol Ther. 2005;11(3):452-9.
